# Iatrogenic pericardial hernia and gastric outlet obstruction

**DOI:** 10.1007/s10554-026-03661-5

**Published:** 2026-02-23

**Authors:** Michael D. Long, Matthew J. Kruse

**Affiliations:** Creighton University School of Medicine, Omaha, Nebraska, USA

**Fig. 1:**
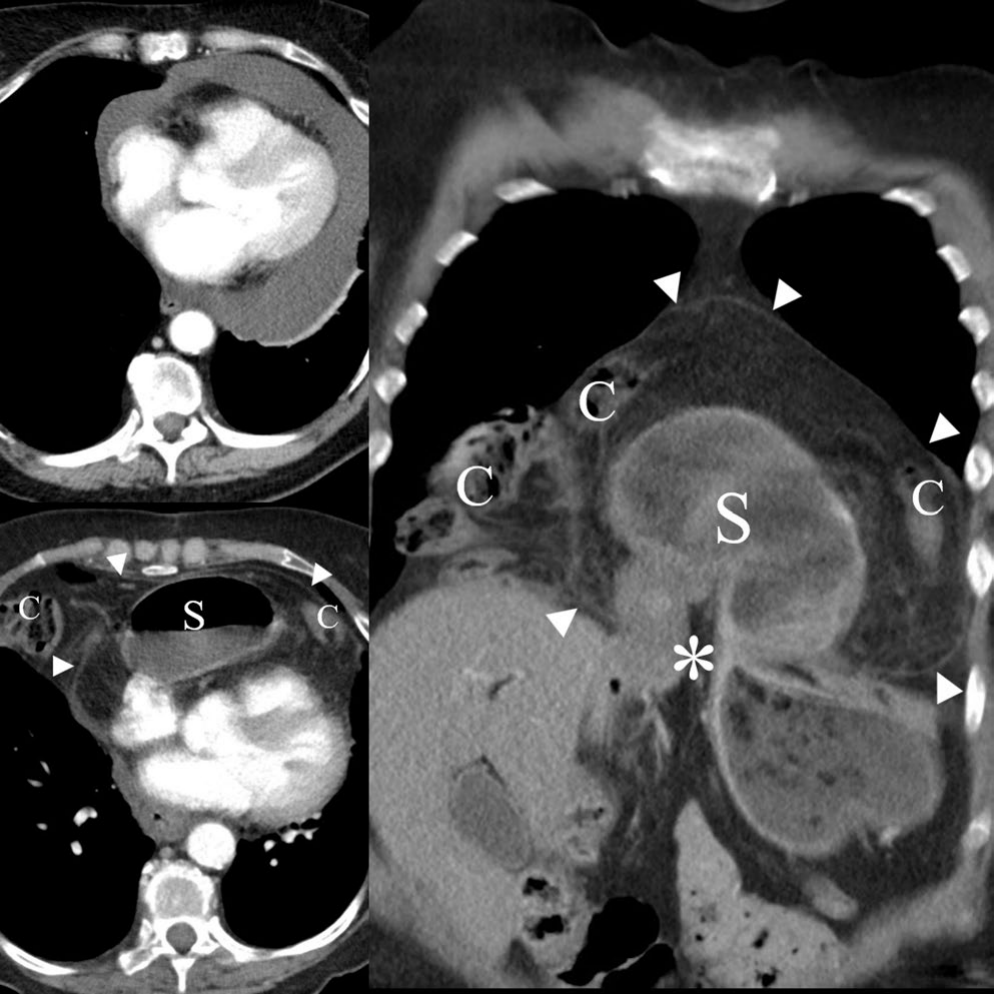
Contrast-Enhanced CT Imaging Before and After Pericardial Window Procedure. Top Left: Axial CT image before pericardial window procedure showing large effusion. Postoperative axial CT image (bottom left) and coronal CT image (right) demonstrate a diaphragmatic hernia (*) into the distended pericardium (arrowheads) at the surgical site, containing the distal stomach (S) as well as colon (C).

Ten weeks after pericardial window procedure, this 67-year-old woman presented with substernal chest pain and intractable emesis. She was diagnosed with gastric outlet obstruction due to gastric antrum herniation into the pericardium, and she was hemodynamically stable despite CT evidence of right heart compression. Management included robotic-assisted diaphragmatic hernia repair with mesh reinforcement. The patient was discharged home after an uncomplicated postoperative recovery.

The pericardial window procedure involves formation of an open channel to allow pericardial fluid to flow into the pleural cavity, performed via a subxiphoid incision. Intrapericardial hernias are exceedingly rare, and risk factors for iatrogenic hernia parallel those of other incisional hernias: obesity, wound infection, and constipation or chronic cough with elevated abdominal pressure [1, 2]. Symptoms include substernal burning, shortness of breath, or cough, with risk of rapid deterioration due to development of tamponade physiology or bowel ischemia [3]. Therefore, immediate identification and management is vital.

## Data Availability

No datasets were generated or analysed during the current study.
